# Content Analysis of Mobile Health Applications on Diabetes Mellitus

**DOI:** 10.3389/fendo.2017.00318

**Published:** 2017-11-27

**Authors:** Syarafina Izahar, Qi Ying Lean, Mohammed Abdul Hameed, Muthu Kumar Murugiah, Rahul P. Patel, Yaser Mohammed Al-Worafi, Tin Wui Wong, Long Chiau Ming

**Affiliations:** ^1^Faculty of Pharmacy, Universiti Teknologi MARA, Puncak Alam, Malaysia; ^2^Faculty of Pharmacy, Universiti Teknologi MARA, Bertam, Malaysia; ^3^Vector borne Diseases Research Group, Pharmaceutical and Life Sciences CoRe, Universiti Teknologi MARA, Shah Alam, Malaysia; ^4^Pharmaceutical Services Division, Penang State Health Department, Georgetown, Malaysia; ^5^Pharmacy, School of Medicine, University of Tasmania, Hobart, TAS, Australia; ^6^College of Pharmacy and Health Sciences, Ajman University, Ajman, United Arab Emirates; ^7^Non-Destructive Biomedical and Pharmaceutical Research Centre, iPROMISE, Universiti Teknologi MARA, Puncak Alam, Malaysia; ^8^School of Pharmacy, KPJ Healthcare University College, Nilai, Malaysia

**Keywords:** diabetes, self-care, health informatics, mobile health, mobile application

## Abstract

Diabetes self-management offers an opportunity to patients to be actively involved in managing their conditions and modifying lifestyle behaviors to attain positive health outcomes. With the unprecedented growth of mobile technology, smartphone plays a role in supporting diabetes self-management. Nonetheless, selecting appropriate mobile applications (apps) is challenging for patients. Thus, this study aimed to evaluate and compare the contents and features of mobile medical apps for diabetes self-management. Of 346 commercial apps, 16 (16%) and 19 (7.72%) of the diabetes apps found in Apple and Google Play stores, respectively, were included based on the selection criteria and individually scored for the availability of 8 main features of diabetes self-management. The apps supported self-management by offering features such as free installation, less than 50 MB space used, offline use, automated data entry, data export and sharing, educational tool, and advice. Of the 8 evaluated features, only 11 (31.4%) apps had a score of 5 whereas 7 (20%) apps scored the lowest, with a score of 3. The majority of apps were free, required no Internet connectivity to use and were less than 50 MB in size. Our findings showed that the design of diabetes mobile apps focused on reporting and setting reminders, rather than providing personalized education or therapeutic support. In the future, the design of apps could be improved to integrate patients’ needs, usability for disease management, and lifestyle modifications.

## Introduction

Diabetes mellitus is often referred to as a type of metabolic disease characterized by a prolonged state of high blood glucose levels in which patients present with symptoms of frequent urination, increased thirst, and hunger (1). As reported by the International Diabetes Federation, in 2015, 415 million of the world’s population have diabetes globally. Approximately 37% of the population (153 million people) live with diabetes in the Western Pacific Region, which is predicted to increase to 215 million by 2040 (2). There were up to 3.3 million cases of diabetes in Malaysia in 2015 (2). Managing patients with chronic diseases is challenging as diabetic patients require knowledge and skills in understanding the needs of medical care, and thus diabetes self-management is crucial, as part of a patient’s commitment to preventing disease complications (1). Diabetes self-management refers to personal actions toward handling the conditions and slowing the progress of the disease (3). Consistent self-monitoring of blood glucose (SMBG) has been shown to improve glycemic control, delaying complications of diabetes, and thus reducing hospitalizations (4). Specifically, diabetes self-management improves health outcome by helping lifestyle modifications including exercise, diet, and medication adherence (5). Apart from being well equipped with knowledge and skills, patients require continuous motivation to effectively cope with diabetes (3), which might necessitate a personalized and comprehensive approach from health-care professionals and health information technology.

Mobile phones have become an essential communication tool globally, the advances in technology have further increased smartphone reliability in various uses (3). Mobile apps are tools which provide various functionalities and services ranging from entertainment, business, education, and self-management, including incorporation into chronic disease management and prevention such as diabetes self-care (6). It has been shown that diabetes self-care can be improved with mobile phone interventions since they offer great potential to support therapy management, to increase therapy adherence, and to prevent disease complications (7). Valuable features of mobile apps have been identified: simple to use, able to provide specific instructions for better disease management, and able to share data with other individuals (4).

Diabetes self-management mobile apps that are designed and developed to manage diabetes may support self-management in diabetes (8). There are various free or paid apps for diabetes yet their use by patients necessitates supervision as the health benefits of mobile apps in managing diabetic conditions are unknown (8). Thus, the use of mobile medical apps which can assist diabetes mellitus management is limited (9). Before mobile apps are promoted to increase their use in managing diabetes among patients, a systematic evaluation of selected mobile medical apps for diabetes mellitus self-management is important to guide diabetic patients or carers in choosing suitable mobile medical apps for diabetes self-management. The evaluation of the contents and features of mobile medical apps for diabetes self-management would allow to rank the usability and completeness of the mobile apps. The evaluation of the contents and features of diabetes mobile medical apps is important as it will provide guidance for diabetic patients in choosing suitable diabetes apps for self-management. The clinicians could also recommend the most suitable mobile apps to their patients and incorporate the mobile apps into the treatment protocol. Therefore, the purpose of this study was to evaluate the contents and the features of mobile medical apps for diabetes self-management. The study might assist mobile app developers in designing new diabetes mobile medical apps or in modifying and improving their existing mobile medical apps.

## Methodology

### Study Design

The search for diabetes mobile medical apps in two platforms: Apple and Google Play stores was done separately by using two smartphones: an iPhone 6 (Apple Inc., Cupentino, CA, USA, for iPhone operating system, iOS device) and a Lenovo A369i (for Android OS device), respectively. The inclusion criteria for the mobile apps were meant to be used for diabetes self-management and have a SMBG, which enables the user to record their blood glucose measurement using the apps. The mobile apps which were specifically designed for medical practitioners rather than patients were excluded from the study. The apps which provided no English-language user interface and/or were developed for use in certain countries other than Malaysia were not included in the evaluation. The apps which offered a general fitness functionality that could be used for diabetes management but were not specifically designed for it were also not part of the study. Apps which were not meant to be used with smartphones were not evaluated, for example, those apps for tablets or iPads only. The apps which were not regularly updated (with a latest updated date of 18 months prior to the study) were also excluded from the study.

**Figure 1:**
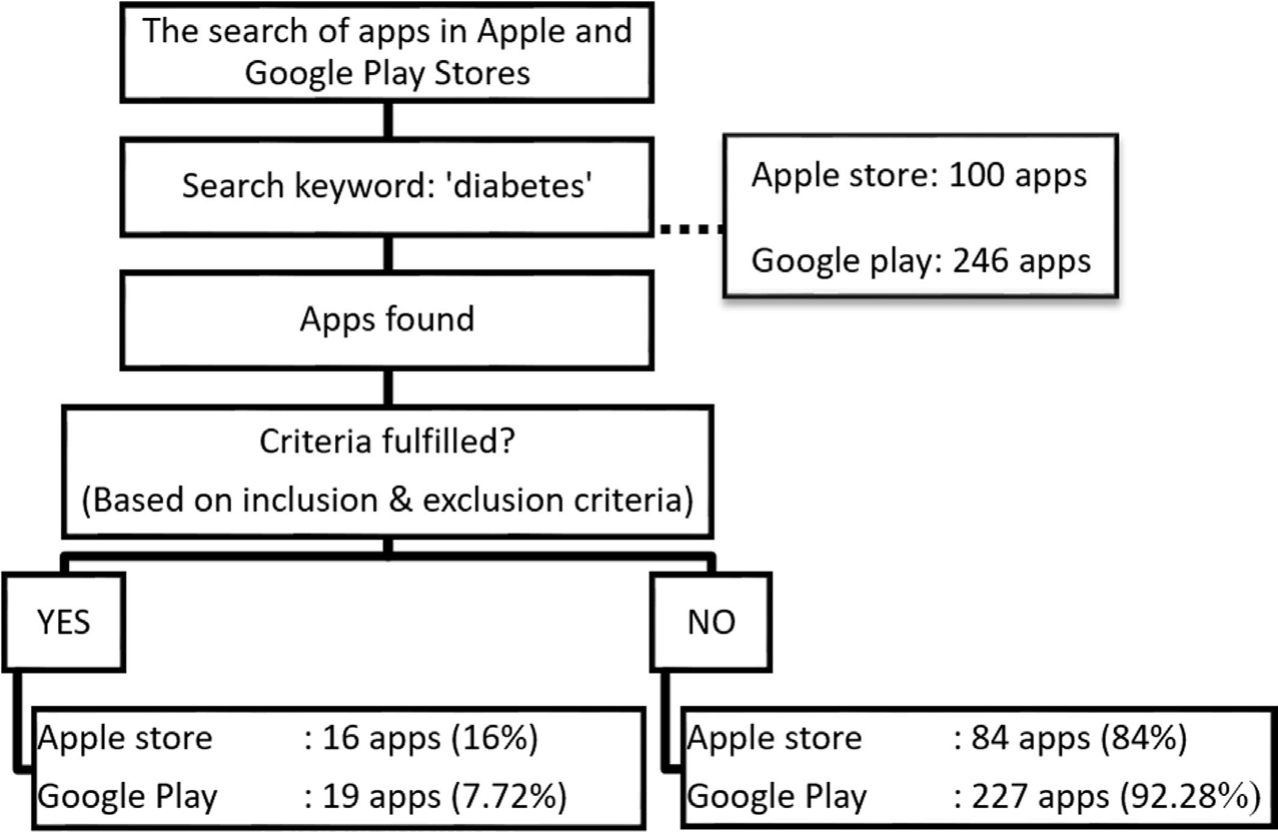
Selection process of mobile medical applications.

First, the mobile apps were searched for using a search keyword “diabetes” (Figure 1). Mobile apps which fulfilled all of the criteria were assessed for the availability of the following features. Based on a literature review (10–13) and discussion among two senior clinical pharmacists and two medical specialists with more than 10 years of clinical experiences managing patients with T2DM, eight selected areas were used for the purpose of comparison among the mobile apps: (i) no Internet requirement, (ii) size of application less than 50 MB, (iii) no subscription requirement, (iv) education tool (diabetes teaching), (v) communication (exporting reports), (vi) automatic data entry option (glucose data can be automatically synchronized into mobile apps’ log data by connecting the glucose meter to mobile), (vii) reminders (remind users to check their blood glucose and take their medications at specific time intervals), and (viii) advisory (therapeutic support). One point was given to the availability of each feature, with a total score of 8.

## Results

**Figure 2:**
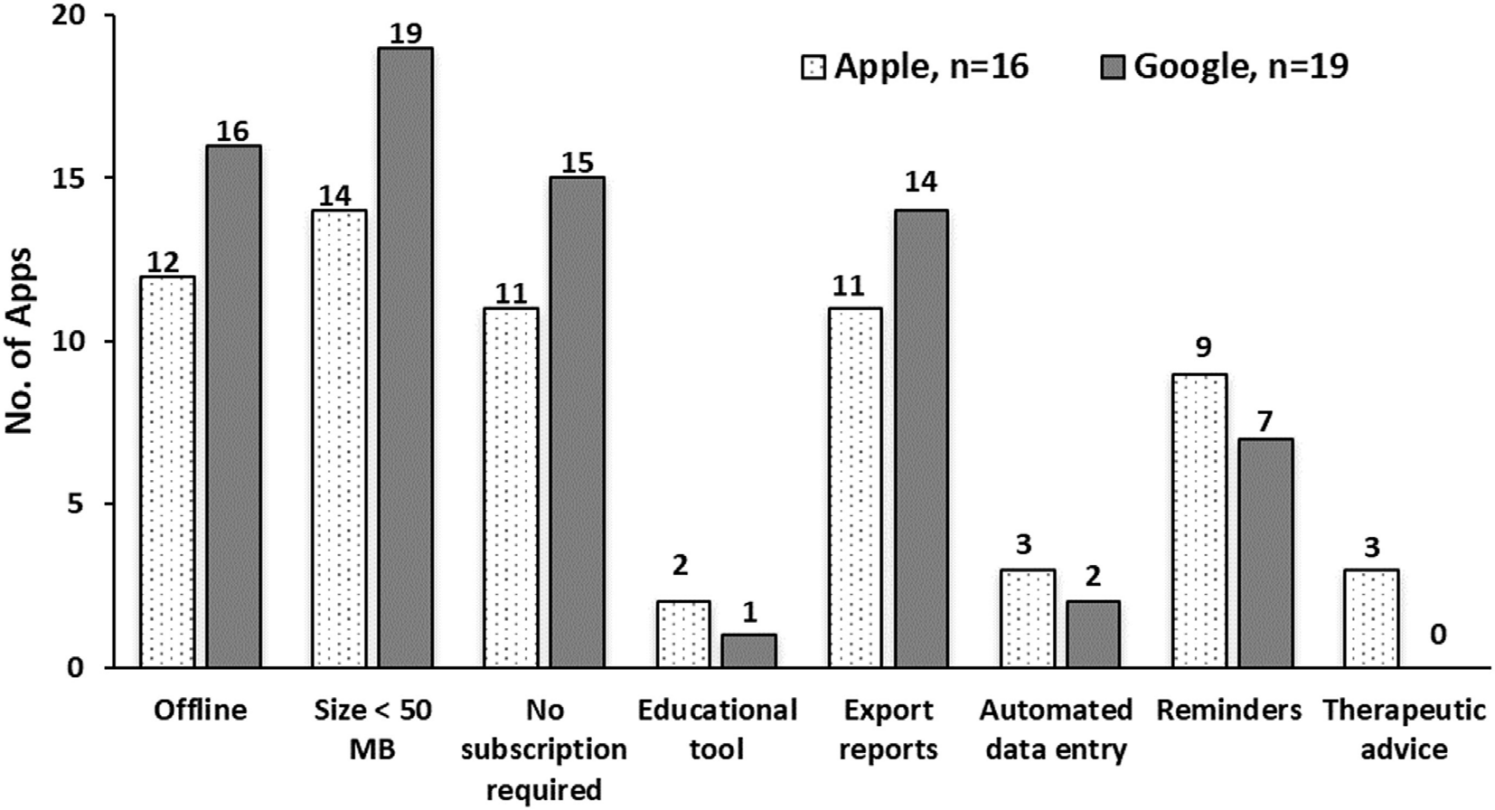
Features of mobile applications available in Apple iPhone operating system and Google Android.

The search for diabetes apps in Apple Apps Store and Google Play yielded a total of 100 and 246 apps, respectively. Only 35 (15.9%) mobile medical apps met the inclusion criteria: 16 (16%) from Apple Store, and 19 (7.72%) from Google Play. Their features are illustrated in Figure 2.

Of the included apps, 87.5% of apps from Apple store and all Android apps from Google Play had the size of less than 50 MB (Figure 2). A slightly higher proportion of Android apps (73.7–84.2%) could be downloaded for free, could be used during offline, and enabled data export compared to apps from the Apple store (68.7–75%). On the other hand, a high proportion of apps from iOS (56.3%) had a reminder feature than apps from the Google Play store (36.8%). The features least possessed by Apple iOS apps were educational tool for learning about diabetes (12.5%), automated data entry (18.75%), and advice (18.75%). A total of three apps from the Apple store provided therapeutic advice as one of their features, while none of the Google Android apps had this feature.

When assessing the features of each app, none of the apps had a score of 6–8 from the total features. Of the 16 Apple mobile apps, 6 apps (37.5%) scored 5. Five apps (31.25%) scored 4, and the remaining apps (*n* = 5, 31.25%) scored 3 (Table 1). From the total of 19 selected mobile apps for Android, only 5 (26.32%) scored 5, while 7 (36.84%) scored 4, and 7 scored 3 (Table 2). Although some apps scored the same, the features they offered varied (Tables 1 and 2).

**Table 1:** Mobile medical apps for Apple iPhone operating system and their features.

No.	Mobile apps’ logo and name, name of developer	Last updated	Features								Total score
			Use offline	Size <50 MB	No subscription required	Educational tool	Export report	Automated data entry	Reminders	Advisory	
1	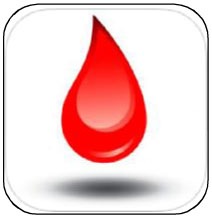 MyGlycemia, by InsyncApp	September 10, 2015	✓	✓	✓		✓		✓		5
2	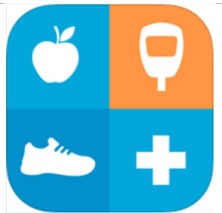 Glooko, by Glooko, Inc.	May 31, 2016	✓				✓	✓	✓	✓	5
3	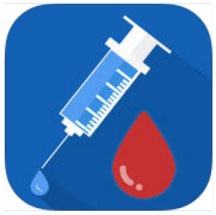 Glucose Companion Free, by Maxwell Software	October 27, 2015	✓	✓	✓		✓		✓		5
4	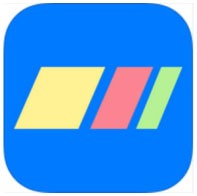 Sugar Streak, by Sugar Streak	April 25, 2016	✓	✓	✓		✓		✓		5
5	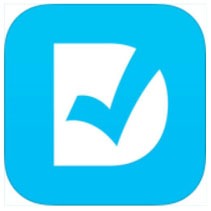 Diabetes in Check, by Everyday Health, Inc.	June 9, 2016			✓	✓	✓		✓	✓	5
6	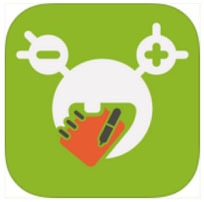 MySugr Diabetes Logbook, by mySugr GmbH	June 5, 2016	✓	✓		✓	✓		✓		5
7	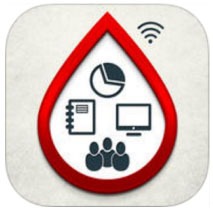 Diabetes Pal App, by Telcare, Inc.	June 1, 2016	✓	✓	✓			✓			4
8	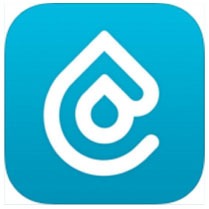 AgaMatrix Diabetes Manager, by AgaMatrix	June 9, 2016	✓	✓	✓		✓				4
9	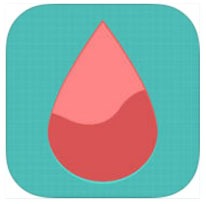 Glucose Wiz, by Linklinks LTD.	May 24, 2016	✓	✓	✓				✓		4
10	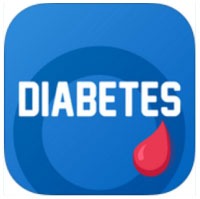 Diabetes Pacer, by Pacer Health, Inc.	January 3, 2015	✓	✓			✓		✓		4
11	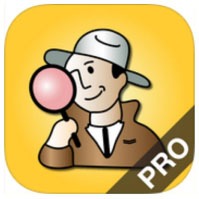 Diabetes Pilot Pro, by Digital Altitudes, LLC	April 28, 2015		✓	✓		✓		✓		4
12	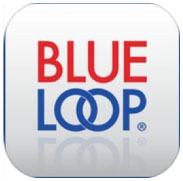 BlueLoop, by BlueLoop, LLC	September 23, 2015		✓	✓		✓				3
13	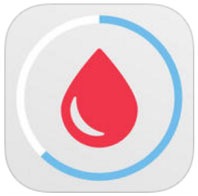 Diabetes Kit Blood Glucose Logbook, by Diabetes Labs, LLC	April 12, 2016	✓	✓	✓						3
14	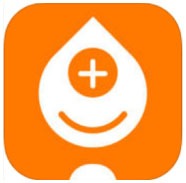 Sugar Sense, by MedHelp	June 8, 2016		✓	✓					✓	3
15	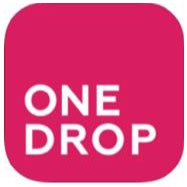 One Drop, by Informed Data Systems, Inc.	May 28, 2016	✓	✓			✓				3
16	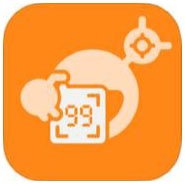 MySugr Scanner, by mySugr GmbH	June 1, 2016	✓	✓				✓			3

**Table 2:** Mobile medical apps for Google Android and their features.

No.	Mobile apps’ logo and name, name of developer	Last updated	Features								Total score
			Use offline	Size <50 MB	No subscription require	Educational tool	Export report	Automated data entry	Reminders	Advisory	
1	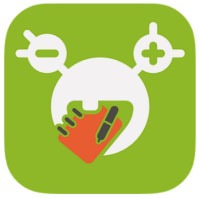 MySugr Diabetes Logbook, by mySugr GmbH	June 10, 2016	✓	✓		✓	✓		✓		5
2	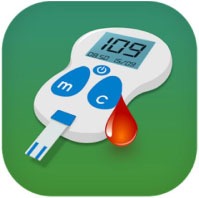 Diabetes Tracker, by Mig Super	December 18, 2015	✓	✓	✓		✓		✓		5
3	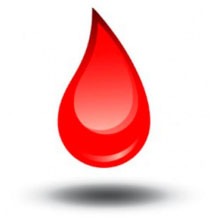 My Glycemia, by InSyncApp	April 12, 2016	✓	✓	✓		✓		✓		5
4	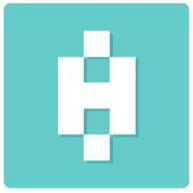 Health2Sync, by H2 Inc.	May 30, 2016	✓	✓	✓		✓	✓			5
5	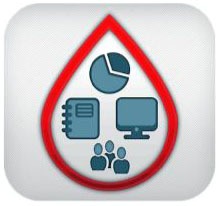 Diabetes Pal, by MyTelcare	May 6, 2015	✓	✓	✓		✓	✓			5
6	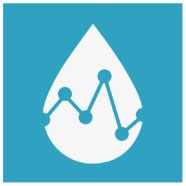 Diabetes:M, by Sirma Medical Systems	March 29, 2016	✓	✓	✓		✓				4
7	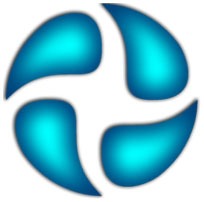 Diabetes, by Klimaszewski Szymon	June 2, 2016	✓	✓	✓		✓				4
8	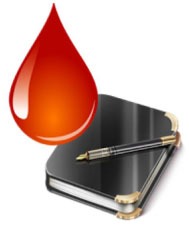 Diabetes Journal, by Suderman Solutions	June 4, 2016	✓	✓	✓		✓				4
9	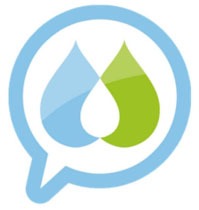 Social Diabetes, by SocialDiabetes	June 1, 2016	✓	✓	✓				✓		4
10	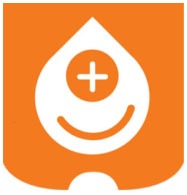 Sugar Sense, by MedHelp	June 6, 2016	✓	✓	✓		✓				4
11	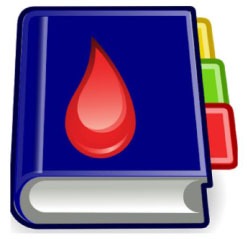 DiaLog, by David Froehlich	February 4, 2016	✓	✓			✓		✓		4
12	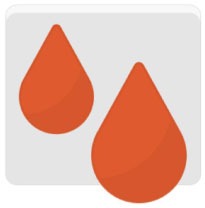 OnTrack, by Medivo	January 29, 2015	✓	✓			✓		✓		4
13	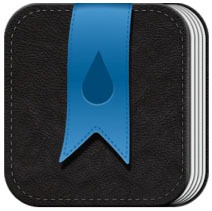 Diabetes Connect, by SquareMed Software GmbH	14 April, 2016		✓	✓		✓				3
14	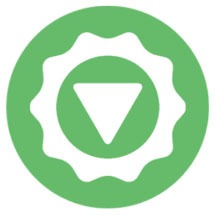 Habits, by Jana Care	May 6, 2016		✓	✓		✓				3
15	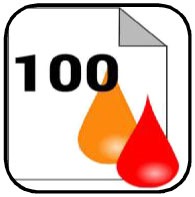 Easy Diabetes, by EasyMobileApp	May 17, 2016	✓	✓	✓						3
16	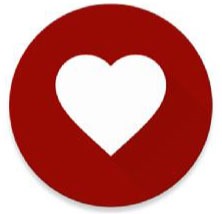 Blood Glucose Tracker, by Little Bytes Software	July 5, 2015		✓			✓		✓		3
17	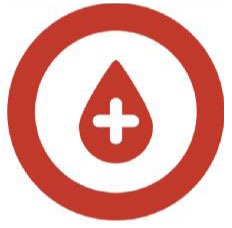 Blood Glucose Manager, by ROOT93 Inc.	May 29, 2016	✓	✓	✓						3
18	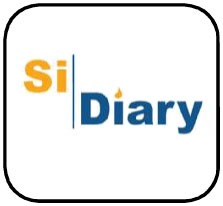 SiDiary, by SINOVO GmbH & Co. KG	March 21, 2016	✓	✓	✓						3
19	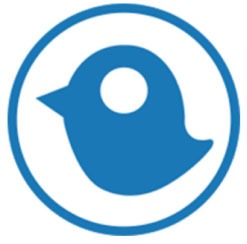 Diabeto, by Diabeto Inc.	February 5, 2016	✓	✓	✓						3

## Discussion

Recent evidence suggest that mobile apps may be utilized to convey health services to patients and can be used as self-management tools (14, 15). In this study, we compared diabetes management apps, either in iOS or Android, with the goal of understanding the content, functionability, and design features. We found that the mobile apps varied in their features and usability. The evaluation of mobile medical apps for diabetes mellitus self-management was conducted through the assessment of the mobile apps’ features. Of 35 apps, the apps with the highest scores of 5 were MyGlycemia, MySugr Diabetes Logbook in both stores; Glooko, Glucose Companion, Sugar Streak, and Diabetes in Check in Apple store; and Diabetes Tracker, Health2Sync, and Diabetes Pal in Google Play store. Most of the apps were relatively small in storage size (<50 MB) and could be used offline. Moreover, most had communication features which enabled the users to export their data files from the mobile phone *via* email (11). This offered patients an electronic system to store their blood glucose records which could be later exported for review during medical follow-up. This was also found to be one of the most favored features for the apps’ users (16).

Nonetheless, there were only a limited number of the apps (for example, Glooko) that offered an automatic data entry feature, which allowed the users to transfer data directly from their blood glucose meter to the phone by cable connection or Bluetooth. Apart from being user friendly, automated data transfer is likely to result in more data captured and reduced errors compared to manual data entry. Glooko is also integrated with other popular health and fitness mobile apps, which allows the users to automatically add physical activities, blood pressure, and body weight data into their health records (17). Moreover, some of the apps such as Glooko and Diabetes in Check provide therapeutic support which enables medical professionals to give feedback based on the data input remotely.

A minority of the apps required a subscription, though this did not mean that they had a particularly high quantity of information, as there were free apps with equal number of features. On the other hand, the reminder feature enables the apps’ users to set the administration time of medications, when to check their blood glucose level, when to take medications, or when to have meals, which might help to improve adherence and prevent episodes of hypoglycemia (18). Practically it may be also useful that the reminder also includes when to visit physicians; for example, once in every 3 months or as often as instructed by the physicians. This simple reminder potentially benefit patients especially elderly who are tend to be forgetful or do not aware of their diabetes conditions. Regular medical follow-ups could ensure patients’ health conditions are under control (1).

Diabetes self-management education is one of the fundamental elements of a comprehensive treatment plan in patients, which ultimately aim to have a positive impact on clinical outcomes. Notably, only three of the reviewed apps had an incorporated educational tool. The majority of reviewed apps did not include this element probably due to the lack of use of clinical guidelines and involvement of health-care teams in preparing patient-oriented health-care apps. To achieve the goals of medical treatment, there is a distinct need to include an educational component as it helps to increase awareness of the importance of adherence and good practices (8). According to the American Association of Diabetes Educators, there are seven key areas to focus on to help patients to manage their diabetes (1), including healthy eating, being active, glucose level monitoring, compliance with medication, cardiovascular risk reduction, and coping with stress. With the development of information technology such as mobile apps, strategies for managing glucose level and other metabolic parameters have become convenient and simple (11). Diabetes mobile apps helps patients to set their goals based on the seven proven diabetes management approaches, thus improving the self-management of diabetes in patients (19). In addition, the main foundation in empowering diabetic patients to effectively manage their diabetes condition is by implementing diabetes self-management education (3). Through the education, patients gain knowledge and skills required to self-manage their diabetes (20). This includes healthy eating and diabetes complication risk reduction behaviors. Recommendations on healthy diet plan for diabetic patients help to improve patients’ nutritional choice, caloric intake, and thus assisting patients’ body weight management and glycemic control. Recently, programs using mobile phone and video gaming have shown to change health behaviors of diabetic patients by providing them with the information on self-care including healthy choice of food and physical activities (21, 22). Therefore, in the aim of training patients with diabetes to self-manage, which could be assisted by the mobile apps, the newly developed digital tools should be comprehensive, incorporating all the abovementioned features. Nonetheless, diabetes self-management with the intervention of smartphones is limited to those patients who are equipped with such a device and who are technology literate. Thus, this could be a major limitation for diabetes self-management among the older patients (23).

Moreover, the majority of apps have not been evaluated for their potential in providing benefits or improving the health outcomes of patients. The selection of the most suitable and satisfactory mobile apps will require patients’ consideration of their personal needs, such as insulin dose and diet modifications (15). Although patients are free to choose the apps, they may not be able to distinguish which is the most helpful, easiest, and effective one to integrate as part of their diabetes care. Patients’ age, the cost of apps, and app-specific features are factors to consider when health-care practitioners help patients to identify useful apps as part of diabetes self-management (4, 8).

To produce quality mobile apps, local experts, international health professionals, or organizations should be involved when adopting evidence-based guidelines into their diabetes care. For instance, it has been shown that patients with diabetes using a mobile software which was designed by endocrinologists, achieved statistically significant improvements in hemoglobin A1c level (24). Thus, it is important to assess the clinical outcome and effectiveness of social media or medical apps to ensure their reliability and adoption as a useful tool in diabetes self-management (25). Comparative or randomized control studies are required to establish clinical evidence of successful patient engagement and cost benefits of mobile medical apps used in patients with diabetes. Meanwhile, points of care should include an introduction on how to incorporate useful mobile apps in managing diabetes in patients. The mobile apps are not only educational and informative but should be carefully designed to assist and facilitate self-management, for the intended users, who are mostly elderly. In addition, patients’ adherence can be improved by implementing strategies of self-monitoring which work best for groups of patients with a specific disease (1).

This study had several limitations. The number of apps for diabetes continues growing rapidly, meaning new apps were available by the time the study had finished. There was no exact download statistics or formal reviews from users to know the preference or effectiveness of each apps. The apps scoring system in this study had not been used in any previous studies. The features covered the specific categories of diabetes self-management yet neither exhaustive nor endorsement of superior quality of each apps. Since, this study also did not assess the quality of the information provided by the apps, thus it could not identify the apps which provide the most evidence-based information. Moreover, an app may offer many features but have poor quality information, and *vice versa*.

## Conclusion

This study shows that there are apps tracking patient medical records especially blood glucose level and setting up reminders, which can improve diabetes control. Mobile apps have great potential in integrating patient self-care education and motivating patients in maintaining healthy behaviors, thus assisting them in managing their chronic diseases. Nonetheless, it is important to ensure the mechanism of integration and appropriateness of mobile apps in assisting patients’ diabetes self-management (13). Research into the delivery of patient-centered mobile apps with reliable and useful information, without compromising user safety and privacy, is desirable to assist diabetes self-management.

## Author Contributions

SI, QL and LM conceived the concept; SI, MH, MM, and QL wrote the initial draft; YA-W, RP, QL, and LM finalized the manuscript. All authors contributed toward revising the paper and agreed to be accountable for all aspects of the work.

## Conflict of Interest Statement

The authors declare that the research was conducted in the absence of any commercial or financial relationships that could be construed as a potential conflict of interest.
